# Head and neck cancer: genetic polymorphisms and folate metabolism

**DOI:** 10.1590/S1808-86942012000100021

**Published:** 2015-10-20

**Authors:** Ana Lívia Silva Galbiatti, Mariangela Torreglosa Ruiz, José Victor Maniglia, Luis Sérgio Raposo, Érika Cristina Pavarino-Bertelli, Eny Maria Goloni-Bertollo

**Affiliations:** a Biologist (Master's degree student in Health Science – Research Unit on Genetics and Molecular Biology (Unidade de Pesquisa em Genética e Biologia Molecular or UPGEM); b Doctoral degree in Health Science (Adjunct professor – Triangulo Mineiro Federal University (Universidade Federal do Triângulo Mineiro or UFTM); c Medical doctor, adjunct professor (Adjunct professor – Sao Jose do Rio Preto Medical School (Faculdade de Medicina de São José do Rio Preto or FAMERP); d Medical doctor (Master's degree student in Health Science); e Adjunct professor of medical and human genetics (Adjunct professor – Sao Jose do Rio Preto Medical School (Faculdade de Medicina de São José do Rio Preto or FAMERP); f Adjunct professor on medical and human genetics (Adjunct professor). FAMERP – Faculdade de Medicina de São José do Rio Preto (Sao Jose do Rio Preto Medical School)

**Keywords:** genes, head and neck neoplasms, polymorphism, genetic

## Abstract

Epidemiological evidence suggests that genetic variants encoding enzymes involved in folate metabolism may modulate HNSCC risk by altering DNA methylation synthesis and genomic estability.

**Aim:**

A review of the literature on genetic polymorphisms involved in folate metabolism and risk of head and neck cancer was carried out.

**Methodology**: An electronic search was made on the Medline database to select papers on head and neck cancer and polymorphisms involved in folate metabolism.

**Results:**

The association between MTHFR C677T polymorphism and the risk of this tumor type was evaluated in nine studies; there was an association with this disease in three papers. The MTR A2756G and MTRR A66G and RFC1 A80G polymorphisms were also associated with increased risk for HNSCC. MTHFD1 G1958A polymorphism was not associated with increased risk of this disease; the evaluation results of the MTHFR A1298C polymorphism in this neoplasm were contradictory. Other polymorphisms involved in folate metabolism were not studied for this neoplasm.

**Conclusion:**

We conclude that polymorphisms involved in folate metabolism may modulate the risk of head and neck cancer, however, these results need to be demonstrated in different populations.

## INTRODUCTION

Head and neck carcinoma is the fifth most frequent cancers; its incidence worldwide has been estimated at 50,000 new cases each year^1^, ^2^. The estimated rate for oral cancer in Brazil during 2010 was 14,120 new cases (10,330 men and 3,790 women)^3^. Most of these epithelial tumors are classified as head and neck squamous cell carcinoma (HNSCC); the anatomical sites and occurrences in this group are the mouth (40%), the pharynx (15%), and the larynx (25%)^2^, ^4^, ^5^, ^6^.

About two thirds of patients with these diseases present at advanced stages in which regional lymph nodes are generally involved. Distance metastases are present in 10% of patients^7^. The treatment varies depending on the stage of the disease; according to published data, about 60% to 65% of head and neck cancer patients may be cured by surgery and/or radiotherapy. Patients at initial stages of the disease (I and II) are treated by a single form of therapy (surgery or radiotherapy), while patients at more advanced stages (III and IV) require a combined approach, such as surgery and radiotherapy or chemotherapy^8^.

This cancer affects mostly males at more advanced age groups; the mean age at diagnosis is 60 years^7^, ^9^. However, the incidence of cancer involving the base of tongue and the tonsils has increased in individuals aged below 45 years; this has been attributed to an increased prevalence of HPV infection, which is a contributing factor for this disease in developing countries^10^, ^11^.

The main and well-established risk factors for this disease are smoking and alcohol abuse; these habits jointly multiply the risk of cancer, especially in the mouth and pharynx^12^. The reason for this is that cigarettes contain about 4,700 substances, of which at least 50 are carcinogenic. Frequent consumption of alcohol renders epithelial cells unable to form a protective barrier against external agents, thereby facilitating the action of cigarette carcinogens, which form DNA adducts that are not recognized in DNA replication processes^13^, ^14^.

Hashibe et al.^12^ published a study showing that alcohol abuse, independently from smoking, elevated significantly the risk of oropharyngeal, hypopharyngeal, and laryngeal cancer in individuals that had never smoked. Alcohol abuse may also cause nutritional deficiencies because of altered intestinal absorption, and may alter important metabolic pathways, such as the folate metabolism, which is involved in cell methylation reactions. Consequently, gene methylation with a potential role in carcinogenesis may be compromised^15^.

Studies have suggested that poor oral hygiene is associated with a higher risk of head and neck cancer. Periodontal disease because of poor oral hygiene may result in infection; inflammation mediators – such as cytokines – are released and reactions against inflammation occur, which may foster the development of cancer^16^. Loss of teeth may also facilitate the onset of mouth cancer, as the oral flora may become abnormal, and nitrite and nitrate reduction and production of acetaldehyde may occur, leading to formation of DNA adducts^14^, ^17^.

Published papers have shows that a diet rich in whole cereals, fruit, and vegetables, and with few processed foods, together with a healthy life style, may confer protection against DNA oxidative damage. These foods contain micronutrients – vitamins B, C, E, carotenoids, flavonoids, and other – that possess antioxidant and anticarcinogenic activity, which reduces the risk of oral cancer^18^, ^19^, ^20^, ^21^.

Folate deficiency in the body – a vitamin that may be found in fruit and vegetables – has been associated with an increased risk of several types of cancer, including head and neck cancer^20^, ^22^, ^23^, ^24^, ^25^, ^26^. This micronutrient is involved in DNA synthesis, repair, and methylation^22^, ^27^.

## OBJECTIVE AND METHODS

The purpose of this study was to carry out a review of the literature to present the results of studies that have assessed the modulation of polymorphisms involved in folate metabolism and the risk of head and neck cancer.

## FOLATE METABOLISM

Folate is involved in forming methyl (CH3) groups during a carbon interconversion in the intermediate metabolism of S-adenosilmethionine (SAM), which is a methyl group donator in cell methylation reactions^26^, ^28^, ^29^. DNA methylation consists of transferring methyl groups to position 5 of cytosine residues that are located on cytosine-guanine dinucleotides (CpG); this occurs in reactions catalyzed by proteins named DNA methyltransferases^30^. This epigenetic DNA modification has several functional roles, such as controlling gene expression, stabilizing chromatin structure, and maintaining genomic stability^23^, ^26^, ^29^, ^30^, ^31^, ^32^, ^33^, ^34^.

There are three mechanisms by which altered folate metabolism may contribute to carcinogenesis: (1) DNA hypomethylation and subsequent proto-oncogene activation^26^, ^35^; (2) uracil misincorporation during DNA synthesis, leading to genomic instability^26^, ^35^, ^36^; and (3) increased cytosine deamination in DNA methylation sites^26^, ^36^.

Abnormal folate levels due to genetic polymorphisms in its metabolic pathway are associated with altered DNA methylation, synthesis and repair; adequate folate levels are essential for the biosynthesis of purines and pyrimidines, which are needed in these biological processes^37^, ^38^, ^39^, ^40^, ^41^, ^42^, ^43^, ^44^, ^45^.

**Figure 1:**
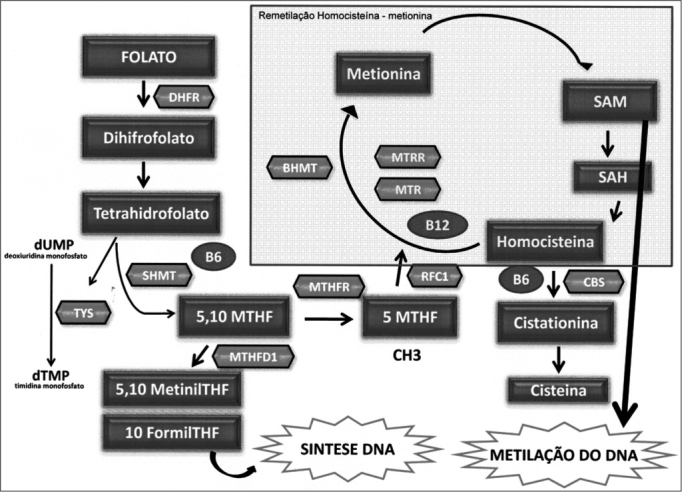
Main enzymes involved in the folate metabolism. Folate metabolism – DHF: Dihydrofolate; THF- Tetrahydrofolate; DHFR: Dihydrofolate reductase; SHMT: Serine hydroxymethyltransferase; TYS: Thymidylate synthase; MTHFD1: Methylenetetrahydrofolate dehydrogenesase 1; MTHFR: Methylene tetrahydrofolate reductase; MTR: methionine synthase; MTRR: Methionine synthase reductase; BHMT: Betaine-homocysteine methyltransferase; CBS: Cystathionine beta synthase; RFC1: Reduced folate carrier 1; SAM: S S-adenosilmethyonine; SAH: S- adenosylhomocysteine; dUMP: Deoxyuridine monophosfate; dTMP: Thymidine monophosfate.

Figure 1 shows the enzymes that are involved in folate metabolism. First, folate is converted or reduced into physiological folate by the dihydrofolate reductase (DHFR) enzyme. The serine hydroxymethyltransferase enzyme (SHMT) catalyzes a reversible reaction of THF into 5,10-MTHF; this enzyme is key in maintaining and regulating the homeostasis of folate concentration and intracellular methyl groups. It requires vitamin B_6_ and has a significant role in protein and DNA synthesis and in methylation reactions involving nucleic acids^46^, ^47^.

The methylenetetrahydrofolate reductase (MTHFR) enzyme catalyzes the conversion of 5,10 methylenetetrahydrofolate into 5- methyltetrahydrofolate (5-MTHFR), which is the main circulating form of folate; it operates as a methyl group donor for the remethylation of homocysteine (Hcy) into methionine. This reaction is catalyzed by the methionine synthase enzyme (MTR), which requires vitamin B_12_ (methylcobalamin) as a cofactor, and which forms SAM. The methionine synthase reductase (MTRR) enzyme maintains the active state of the MTR enzyme. Following the methylation of Hcy, the resulting methionine is condensed with adenosine triphosphate (ATP), which results in S-adenosilmethionine (SAM). Next, in a demethylation reaction, S-adenosylhomocysteine (SAH) is formed and then hydrolyzed to release adenosine and Hcy, thereby completing the cycle^48^.

Hcy methylation replenished the stocks of SAM when methionine reaches lower levels. The betaine homocysteine methyltransferase (BHMT) enzyme catalyzes the conversion of Hcy into methionine by an alternative remethylation pathway in which the betaine amino acid donates the methyl groups^49^, ^50^, ^51^. When the Hcy remethylation pathway – which is catalyzed by the folate-dependent MTR enzyme – is altered by genetic or environmental factors, the BHMT enzyme has a crucial role in Hcy homeostasis^52^.

Also involved is the cystathionine b-synthase (CbS) enzyme (also requiring vitamin B_6_), which has a crucial role in folate metabolism; it converts Hcy into cystathionine in the transsulfuration pathway^53^, ^54^.

THF is recovered during the methionine regeneration cycle, after the methyl group (5-metil -THF) is donated to homocysteine. THF may be used directly – in another pathway – in the synthesis of thymidylate synthase (TS), which converts deoxyuridine monophosphate (dUMP) into thymidine monophosphate (dTMP) by using the 10-formyl-THF for DNA synthesis. In this reaction, 5,10 methylene THF is the substrate of thymidylate^55^.

The methylenetetrahydrofolate dehydrogenase 1 (MTHFD1) enzyme catalyzes the oxidation of 5,10-methylene-THF into 5,10-methynyl-THF, which is then converted into 10-formyl-THF (Stevens *et al.,* 2007). These three reactions are involved in the interconversion of THF carbon-1 derivates, which are the substrates for synthesizing methionine, thymidylate, and purines^56^.

Another enzyme is the reduced folate carrier 1 (RFC1) enzyme, which is found on the membrane of intestinal mucosa cells, and which is involved in folate absorption. It does so by transporting 5-MTHF into several types of cells, and is an important determinant of folate concentration within cells^48^.

## GENETIC POLYMORPHISMS INVOLVED IN FOLATE METABOLISM AND HEAD AND NECK CANCER

### The *MTHFR* C677T polymorphism

This polymorphism is associated with decreased enzyme activity by limiting conversion of 5,10 methylenetetrahydrofolate into 5-MTHFR, which is the folate form required for DNA methylation reactions^57^. A in vitro study has shown that the heterozygous 677CT genotype is associated with a 40% decrease in enzyme activity, while the polymorphic homozygote 677TT genotype is associated with a 70% decrease in enzyme activity^58^.

Additionally, the polymorphic homozygote genotype is associated with lower folate levels and higher homocysteine levels in blood plasma^59^, ^60^, ^61^; thus, reduced plasma folate levels may lead to hypomethylation of DNA and cancer^62^.

**Chart 1:**
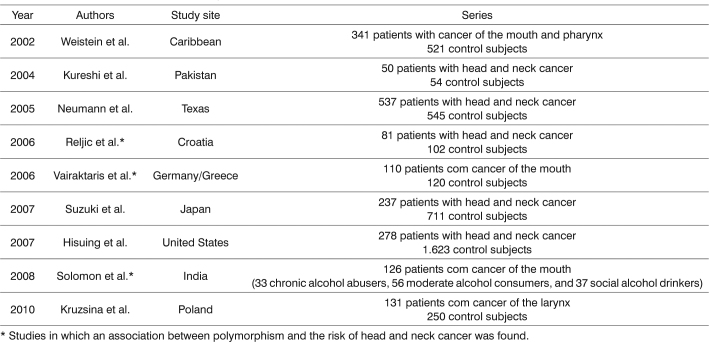
Studies on MTHFR C677T polymorphism in head and neck cancer.

^*^Studies in which an association between polymorphism and the risk of head and neck cancer was found.

As far as we know, eight studies have assessed the association of this polymorphism with head and neck cancer^37^, ^38^, ^39^, ^40^, ^41^, ^42^, ^43^, ^44^. Of these, only Reljic et al.'s^41^, Vairaktaris et al.'s^40^, and Solomon et al.'s^43^ papers have confirmed an association of the MTHFR C677T polymorphism with a risk of head and neck cancer (Chart 1).

Reljic et al^41^. conducted a case-control study of 81 patients with head and neck cancer and 102 subjects without a history of cancer among a Croatian population and found that the 677TT genotype decreases the risk of this disease. On the other hand, Vairaktaris et al.^40^ studied 110 subjects with mouth cancer and 102 cancer-free individuals among Germans and Greeks and found that the 677CT genotype was associated with an increased risk of this cancer. Solomon et al.^43^ assessed 126 individuals who were alcohol abusers (33 chronic and significant consumers of alcohol, 56 moderate consumers of alcohol, and 37 social drinkers) and who had mouth cancer and found that the 677TT genotype was associated with the group of chronic and significant alcohol abusers and the group of moderate consumers of alcohol (to a lesser degree).

### The *qMTHFR* A1298C polymorphism

This variant has also been associated in vitro with decreased enzyme activity, although to a lesser degree compared to the MTHFR C677T polylmorphism^63^. A clear biological relevance of this polymorphism remains unclear and results so far are inconsistent^62^, ^64^, ^65^.

Data on the risk of head and neck cancer in relation to the *MTHFR* A1298C polymorphism is contradictory. Suzuki et al.'s^42^ case-control study of 237 Japanese patients with head and neck cancer and 711 cancer-free individuals and Kruzsina et al.'s^44^ study of 131 Polish subjects with laryngeal cancer and 250 cancer-free Polish individuals found no association between this variant and the risk of head and neck carcinoma.

Neumann et al.'s^39^ study of 537 patients with head and neck cancer and 545 control subjects in Texas showed that individuals with the 1298AC or 1298CC genotypes had a 35% lower risk of head and neck cancer. However, this study showed that the risk of HNSCC was higher in individuals with the three polymorphic alleles (*MTHFR* 677T, *MTHFR* 1298C, and *MTHFR* 1793A) compared to subjects with one or two polymorphic alleles.

### The *MTR* A2756G polymorphism

As far as we know, there are no in vitro studies that have assessed the activity of the MTR enzyme in the presence of the *MTR* A2756G polymorphism. Data on changes in Hcy and folate levels are contradictory^66^, ^67^, ^68^, ^69^, ^70^, ^71^. A few authors have shown that individuals with the polymorphic homozygote *MTR* 2756GG genotype present low Hcy and high folate levels^68^, ^69^, ^70^, ^71^. On the other hand, Li et al.^66^ showed that Hcy levels are high if this variant is present, while Ma et al.^67^ showed that this polymorphism does not change Hcy levels.

Studies of DNA methylation have shown that the *MTR* 2756AG or GG genotypes decrease the formation of SAM, which results in DNA hypomethylation^72^. Other studies have also shown that there is a relation between the *MTR* 2756GG genotype and DNA hypomethylation in colorectal, breast, lung, and cervix cancers^72^, ^73^, ^74^, ^75^.

Three studies have demonstrated the association between this polymorphism and head and neck cancer. Zhang et al.^75^ conducted a case-control study in Texas of 721 patients with head and neck cancer and 1,234 individuals with no history of cancer and found that the *MTR* 2756AG or GG genotypes increased the risk of cancer. Kruzsina et al.'s^44^ study of 131 Polish subjects with laryngeal cancer and 250 controls also showed that these genotypes (*MTR* 2756GG or AG) were associated with this tumor type. Our group studied 236 Brazilian patients with head and neck cancer and 469 controls and found that the *MTR* 2756GG genotype and the *MTR* 2756G allele were associated with an increased risk of HNSCC^45^. On the other hand, Suzuki et al.'s^42^ study of 237 Japanese patients with head and neck cancer and 711 controls revealed no association between this polymorphism and head and neck cancer.

### The *MTRR* A66G polymorphism

Studies have shown that this variant generates an enzyme with low affinity for the MTR enzyme^76^. Gaughan et al.'s^77^ study showed that individuals with the *MTRR* 66GG genotype had low levels of Hcy in the blood plasma compared with individuals having the *MTRR* 66AA genotype. This effect, however, was not observed in other studies^78^, ^79^.

Few studies have investigated an association of the *MTRR* A66G polymorphism and the risk of head and neck cancer. Suzuki et al.^42^ showed that this variant is not associated with a risk for head and neck cancer, but these authors also found an interaction between alcohol abuse and the *MTRR* A66G polymorphism in a Japanese population. Zhang et al.^75^ showed that individuals with the homozygous wild genotype (*MTRR* 66AA) are at a lower risk for head and neck cancer, confirming that the A allele is protective.

### The *RFC1* A80G polymorphism

The *RFC1* gene is involved in intracellular folate transport; it causes 5-MTHFR to be absorbed and transported into several cell types. The *RFC1* A80G polymorphism may be involved in carcinogenesis by altering the concentration of plasmatic Hcy and folate, which in turn are associated with DNA methylation and repair. However, the exact biological mechanism of this polymorphism is not clear^48^, ^80^, ^81^, ^82^, ^83^.

Only our group evaluated the *RFC1* A80G variant and its risk for head and neck cancer; we confirmed that the *RFC1* 80AG or 80AA genotypes were associated with an increased risk for these cancers, especially in males aged over 50 years that smoked cigarettes^84^.

### Other polymorphisms of the folate metabolism

Only one study assessed the effect of the *MTHFD1* G1958A polymorphism in head and neck cancer; it found no associated risk for this disese^44^. Associations between head and neck cancer and the *CBS* 844ins68, *BHMT* G742A, *SHMT* C1420T, *TC2* A67G, and *TC2* C776G polymorphisms, which are also involved in folate metabolism, have not been studied; two of these polymorphisms have been associated with other types of cancer^85^, ^86^, ^87^, ^88^, ^89^.

## CONCLUSION

The *MTHFR* C677T*, MTHFR* A1298C, *MTR* A2756G, *MTRR* A66G, and *RFC1* A80G polymorphisms appear to modulate the risk of head and neck cancer. However, because of contradictory findings, studies of different populations are needed to clarify the role of these polymorphisms in the etiology of head and neck cancer.
